# Point-of-care Lung Ultrasound Is More Sensitive than Chest Radiograph for Evaluation of COVID-19

**DOI:** 10.5811/westjem.2020.5.47743

**Published:** 2020-06-19

**Authors:** Joseph R. Pare, Ingrid Camelo, Kelly C. Mayo, Megan M. Leo, Julianne N. Dugas, Kerrie P. Nelson, William E. Baker, Faizah Shareef, Patricia M. Mitchell, Elissa M. Schechter-Perkins

**Affiliations:** * Boston University School of Medicine, Department of Emergency Medicine, Boston, Massachusetts; † Boston Medical Center, Department of Emergency Medicine, Boston, Massachusetts; ‡ Boston University School of Medicine, Department of Pediatric Infectious Diseases, Boston, Massachusetts; § Boston University School of Public Health, Department of Biostatistics, Boston, Massachusetts

## Abstract

**Introduction:**

Current recommendations for diagnostic imaging for moderately to severely ill patients with suspected coronavirus disease 2019 (COVID-19) include chest radiograph (CXR). Our primary objective was to determine whether lung ultrasound (LUS) B-lines, when excluding patients with alternative etiologies for B-lines, are more sensitive for the associated diagnosis of COVID-19 than CXR.

**Methods:**

This was a retrospective cohort study of all patients who presented to a single, academic emergency department in the United States between March 20 and April 6, 2020, and received LUS, CXR, and viral testing for COVID-19 as part of their diagnostic evaluation. The primary objective was to estimate the test characteristics of both LUS B-lines and CXR for the associated diagnosis of COVID-19. Our secondary objective was to evaluate the proportion of patients with COVID-19 that have secondary LUS findings of pleural abnormalities and subpleural consolidations.

**Results:**

We identified 43 patients who underwent both LUS and CXR and were tested for COVID-19. Of these, 27/43 (63%) tested positive. LUS was more sensitive (88.9%, 95% confidence interval (CI), 71.1–97.0) for the associated diagnosis of COVID-19 than CXR (51.9%, 95% CI, 34.0–69.3; p = 0.013). LUS and CXR specificity were 56.3% (95% CI, 33.2–76.9) and 75.0% (95% CI, 50.0–90.3), respectively (p = 0.453). Secondary LUS findings of patients with COVID-19 demonstrated 21/27 (77.8%) had pleural abnormalities and 10/27 (37%) had subpleural consolidations.

**Conclusion:**

Among patients who underwent LUS and CXR, LUS was found to have a higher sensitivity than CXR for the evaluation of COVID-19. This data could have important implications as an aid in the diagnostic evaluation of COVID-19, particularly where viral testing is not available or restricted. If generalizable, future directions would include defining how to incorporate LUS into clinical management and its role in screening lower-risk populations.

## INTRODUCTION

Novel coronavirus, SARS-CoV-2, is responsible for causing the coronavirus disease 2019 (COVID-19). With an estimated case fatality rate of 1%, COVID-19 has resulted in over 305,000 deaths worldwide to date.^1^ COVID-19’s mortality is primarily due to lung injury resulting in acute respiratory distress syndrome (ARDS).^2^ The definition of ARDS has changed over time; however, using the 2012 Berlin definition it would include acute bilateral lung injury in the absence of fluid overload, causing hypoxemia and respiratory failure.^3^ Physicians evaluating patients may wish to order radiographic imaging to screen for findings of COVID-19, evaluate severity of pulmonary involvement, or assess for alternative etiologies of illness. Radiographic results may alter the treating physician’s concern for COVID-19 thereby guiding patient counseling, or supporting clinical choices such as hospitalization, the need for closer follow-up, or anticipating complications of the disease. The American College of Radiology (ACR) recommended the use of portable chest radiograph (CXR) when medically necessary for patients with suspected or known COVID-19, which does not include screening purposes.^4^ However, it is estimated that portable CXR is only 69% sensitive for findings of COVID-19.^5^

When compared to CXR, lung ultrasound (LUS) may offer improved diagnostic accuracy in the evaluation of patients with suspected COVID-19 pneumonia. LUS has a high sensitivity and often out-performs CXR in the diagnosis of other pulmonary infections.^6^ LUS findings for COVID-19 have been reported in the literature and include B-lines, pleural abnormalities, and subpleural consolidations.^7^–^9^ Evaluation of B-lines is already within the scope of practice for emergency physicians (EP), and instruction in interpreting LUS is part of current residency education standards.^10^

### Importance

LUS is a safe, readily available tool that can be employed by EPs to provide real-time clinical assessment for COVID-19. Lab testing utility is hampered by delays in results, accuracy, and availability. CXR may miss pulmonary disease, and the ACR has cautioned against routine screening with chest computed tomography (CT), citing concerns of poor specificity of ground-glass opacities for COVID-19 as well as infection control procedures necessary to decontaminate the CT scanner.^4^ Regarding infection control procedures, we expect that portable (or hand-held) ultrasounds would be easier to decontaminate than portable CXR machines or CT suites.

### Goals of This Investigation

Our primary aim was to determine whether detection of B-lines on LUS, among patients without alternative etiologies for their presence, is more sensitive for the diagnosis of COVID-19 than CXR. Our secondary aim was to evaluate the proportion of patients with COVID-19 that have secondary LUS findings of pleural abnormalities and subpleural consolidations.

## METHODS

### Study Design and Setting

This was a retrospective, observational, cohort study of patients undergoing COVID-19 testing (based on real-time reverse transcriptase-polymerase chain reaction [RT-PCR] of nasopharyngeal sampling performed on an assay developed by the Center for Regenerative Medicine at Boston University, operating under an Emergency Use Authorization], who also had both diagnostic LUS and CXR for the evaluation of COVID-19 in the emergency department (ED). This study had institutional review board approval and was conducted based on Standards for Reporting of Diagnostic Accuracy Studies (STARD) guidelines and best practices for retrospective reviews.^11^

**Population Health Research Capsule**

What do we already know about this issue?

*Lung ultrasound (LUS) has been shown to outperform chest radiograph (CXR) in its ability to detect abnormalities with non-coronavirus disease 2019 (COVID-19) pulmonary infections*.

What was the research question?

*To determine if B-lines detected by LUS are more sensitive for the associated diagnosis of COVID-19 than an abnormal CXR*.

What was the major finding of the study?

*B-lines detected by LUS were more sensitive for the associated diagnosis of COVID-19 than an abnormal CXR*.

How does this improve population health?

*In locations where viral testing is not available or has significant delays, LUS may provide important information for the evaluation of suspected COVID-19*.

This investigation was performed at a large urban academic ED in the United States with >140,000 visits per year. The ED is associated with an emergency medicine residency and clinical ultrasound fellowship, and has six dedicated portable ultrasound machines (Philips SPARQ, Wayne, PA; and MINDRAY TE7, Arnold, MD). All ultrasound studies are transferred wirelessly and stored in QPATH (Telexy, Blaine, WA). There was no formal education for LUS specific to COVID-19; however, all physicians have had structured training in LUS. All physicians were provided literature from a small study of 20 patients with COVID-19 that had 12 lung zones evaluated with ultrasound, which found 75% of patients had abnormal LUS findings at the posterior lung bases.^9^ When performing point-of-care ultrasound in the clinical setting, all EPs at our institution are required to archive at least one image that is representative of their findings.

### Selection of Participants

All ultrasound studies completed in the ED between March 20, 2020–April 6, 2020, were reviewed for LUS imaging. We reviewed the electronic health record (EHR), EPIC (Verona, WI) to determine whether COVID-19 testing was performed. Subjects were included for evaluation if they had a COVID-19 test performed during the index hospitalization or within two weeks of the LUS examination. At the hospital during this time period, COVID-19 testing was performed only on people with symptoms concerning for disease, and no routine screening practices were in place. However, performance of viral testing was at physician discretion, and those without viral testing were excluded from analysis. We also excluded subjects if they did not have a CXR. Lastly, based on EHR review from patient history or physician documentation, patients were excluded if they had reasons for alternative causes of B-lines (congestive heart failure, renal disease leading to volume overload, or underlying lung disease), as it would not be possible to determine the etiology of the abnormal ultrasound results.

### Test Methods

All lung ultrasounds were reviewed by two expert EPs, both with clinical ultrasound fellowship training (JRP and KCM), who were blinded to COVID-19 results. When disagreements occurred, a third ultrasound fellowship-trained, blinded independent expert reviewer adjudicated (MML). LUS were scored as positive or negative after review of all images. Subjects were considered to have a positive LUS if any B-lines were detected. The reviewers further graded positive ultrasounds as having 1–2 B-lines or ≥3 B-lines.^12^ If B-lines coalesced, the score was graded as ≥3 B-lines if the area of B-lines took up ≥30% of the intercostal space. Although ground-glass opacities can manifest as thinner B-lines <3mm apart, we allowed for percentage grading to account for coalescing in addition to “light beam” artifact, which is a broader, band-shaped artifact described in COVID-19.^13^ Because COVID-19 is reported to cause focal and diffuse lung disease, we chose the image with the most B-lines detected at one intercostal space to score each patient.

**Figure 1:**
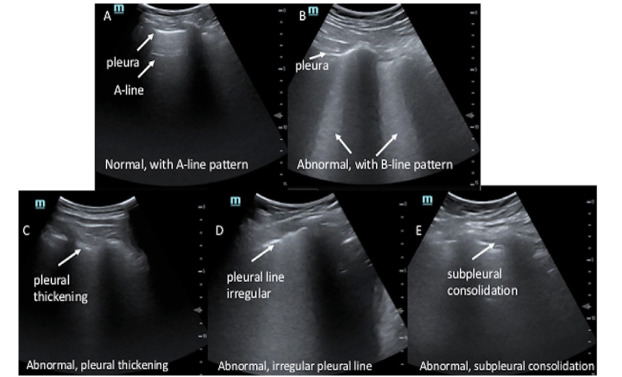
Lung ultrasounds. (A) Normal lung ultrasound. A-lines are horizontal lines that can be seen in the absence of pathology. (B) Abnormal lung ultrasound. The pleura is noted at the top of the lung. This is an example of coalescing B-lines shown as what appear to be headlights coming down from the pleura. (C) Abnormal lung ultrasound. Demonstrated is pleural thickening, >3 millimeters by visual estimate was considered abnormal. (D) Abnormal lung ultrasound. Demonstrated is an irregular pleural line seen in viral infections. (E) Abnormal lung ultrasound. Shown is a subpleural consolidation that appears black between the pleura above the pleural line.

The images were subsequently evaluated for subpleural consolidations and pleural abnormalities (Figure 1 and Online Supplemental Videos A–E). We defined subpleural consolidations as an area of hypoechoic focus at the pleural line. These areas may be associated with increased B-lines originating from this area of hypoechoic focus. For pleural abnormalities we defined this as a) loss of pleural line echogenicity; b) irregular contour of the pleural line; or c) areas that appeared >3 millimeters in thickness by visual estimation.^14^ Secondary LUS findings were determined by a consensus of all reviewers. Finalized CXR reports were recorded. We classified CXRs as positive if the report included infection in the differential, as defined by words such as opacity, consolidation, or airspace disease. CXRs were classified as negative if no abnormality was noted, an abnormality was noted but attributed to a non-infectious etiology, or was inconclusive for infectious process.

After LUS scoring and data collection, clinical data including demographics, co-morbidities, vital signs, and laboratory values, was collected from the EHR by two investigators (JRP and FS) using a standardized abstraction technique and entered into REDCap.

### Outcome Measures

The primary outcome measure was the sensitivity of LUS compared to CXR for the detection of COVID-19, using the RT-PCR laboratory test as the reference standard. Secondary outcome measures were the proportion of additional secondary LUS findings (pleural abnormalities or subpleural consolidation) detected.

### Analysis

A sample size of 43 patients with an estimated sensitivity of 40% for CXR and 70% for LUS yields 81% power with an alpha of 0.05 assuming 70% disease prevalence. We used an estimated sensitivity of 40% based on results of CXR findings in influenza, as the referenced paper of 69% was not available at the time this study was designed.^5^,^15^ We compared sensitivities of LUS and CXR using a two-sided McNemar’s test. Patient demographics were evaluated with descriptive statistics, Fisher’s exact tests, Wilcoxon sum-ranked test, chi-squared tests, and Welch’s t-test. Inter-rater reliability for the primary outcome between the two primary reviewers was assessed by Cohen’s kappa.^16^ In addition, 95% Agresti-Coull confidence intervals (CI) were calculated for CXR and LUS test characteristics. We performed all analyses using SAS v9.4 (SAS Institute Inc., Cary, NC). Sample size calculations were conducted using PASS 19 (PASS 2019 Power Analysis and Sample Size Software (2019). NCSS, LLC. Kaysville, UT).

## RESULTS

### Characteristics of Study Subjects

**Figure 2:**
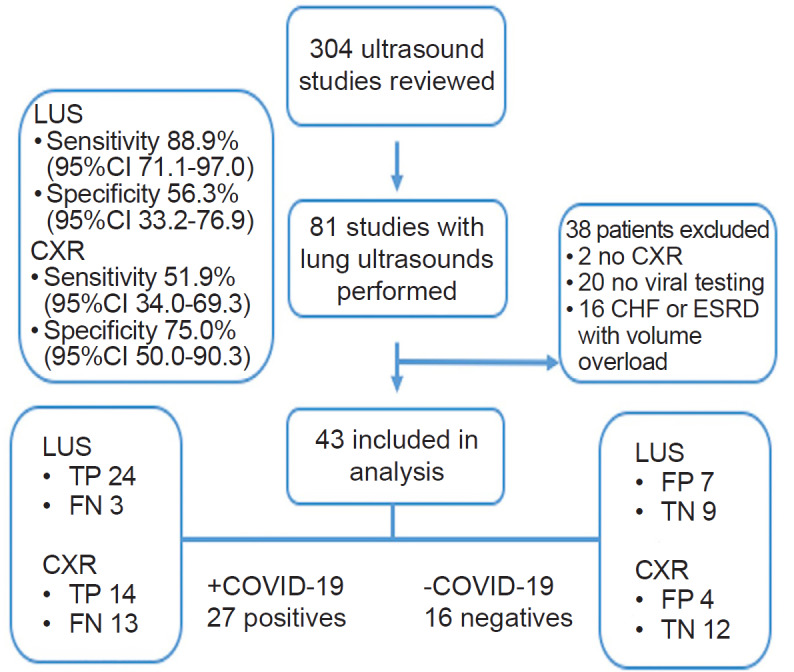
Flow chart of enrollment in lung ultrasound study. *CI*, confidence interval; *CXR*, chest radiograph; *LUS*, lung ultrasound; *CHF*, congestive heart failure; *ESRD*, end-stage renal disease; *TP*, true positive; *FP*, false positive; *TN*, true negative; *FN*, false negative.

A total of 304 ultrasound studies were completed over the 18-day study period (Figure 2). Of these, 81 had LUS performed. Among these, 43 met inclusion criteria, and 27/43 tested positive for COVID-19 by RT-PCR (63%). Four patients admitted with initial negative results were retested, and two were found to be positive. These two subjects were classified in the 27 total patients with COVID-19. Table 1 describes the demographic and clinical information of the included patients.

### Main Results

The sensitivity and specificity of B-lines on LUS associated with COVID-19 were 88.9% (95% CI, 71.1–97.0) and 56.3% (95% CI, 33.2–76.9), respectively. The association between CXR and COVID-19 results had a sensitivity and specificity (Appendix) of 51.9% (95% CI, 34.0–69.3) and 75.0% (95% CI, 50.0–90.3). LUS was more sensitive than CXR for the association of pulmonary findings of COVID-19 (p = 0.013). While there was a trend for CXR to be more specific for the associated diagnosis of COVID-19, this was not found to be statistically significant (p = 0.453). Additional LUS test characteristics are provided in Table 2. Cohen’s kappa for inter-rater agreement between the two expert LUS reviewers for the primary outcome was strong (κ = 0.83, 95% CI, 0.65–1.00). There were only three cases out of 43 where there was disagreement on the primary outcome between the two reviewers. These involved cases where B-lines were more subtle.

B-lines were more frequently detected in patients with COVID-19 (24/27 patients with COVID-19 and 7/16 patients without, p < 0.001). Of the 27 patients with confirmed COVID-19 infection, 21 had pleural abnormalities (77.8%) and 10 had subpleural consolidations (37%). Of the 16 subjects without COVID-19, three had pleural irregularities (18.8%) and two had subpleural consolidations (12.5%).

There was a mean of 6.2 LUS images recorded per patient, which was not significantly different between COVID-19 results, and a median of 6 LUS images taken per patient. Images were more frequently obtained with a curvilinear probe 37/43, (86%), than the phased array probe, 6/43 (14.0%). Of the LUS studies, 8/43 (18.6%) were completed by residents or physician assistants, 4/43 (9.3%) by an ultrasound fellow, 17/43 (39.5%) by ultrasound faculty, and 14/43 (32.6%) by non-fellowship trained EPs. Of the CXRs performed, 42/43 (97.7%) were performed as portable examinations. The one 2-view CXR was a false negative.

## DISCUSSION

To our knowledge this is the first study to evaluate the test characteristics of LUS for COVID-19. We also are the first to compare the diagnostic performance of LUS to the more conventional use of CXR. Although preliminary, this work provides important results for the application of LUS for detection of COVID-19. This investigation offers compelling evidence that B-lines detected by LUS are more frequently associated with COVID-19 than an abnormal CXR. This finding is in line with the performance of LUS in other pulmonary disease entities.^6^,^10^

We used RT-PCR as the reference standard for diagnosis of COVID-19. However, it is known that the test characteristics of RT-PCR are dependent on collection technique, timing in disease process, and processing technique. In our population there were two negative RT-PCR tests that were positive on repeat testing. Both patients with initially negative RT-PCR tests had positive LUS findings; thus, it is possible LUS is more sensitive than RT-PCR for COVID-19. Further research would be necessary to substantiate this theory.

Our study reports a sensitivity of 52% for CXR, which is lower than the reported 69% for portable CXR. It is unknown whether the radiologists in that previous study were blinded, and it is also unclear how body mass index or other variables may have resulted in our reported lower sensitivity for CXR. It is unknown how two-view CXRs would perform for the detection of lung involvement from COVID-19, as it might outperform portable CXR. However, given the infectious nature of COVID-19 portable CXR is the recommended diagnostic test for patients with suspected COVID-19, and these results demonstrate a generally low sensitivity.

Evidence that LUS is more sensitive for the associated diagnosis of COVID-19 than CXR has potential global implications. These results may be of particular importance to settings with significant delays in viral RT-PCR testing, settings in which RT-PCR testing is restricted or not available, or where CXR or CT are not accessible. Further scientific investigation could determine how LUS at the time of initial evaluation may aid the physician in counseling patients with regard to findings suggestive of COVID-19. Our investigation provides important new data for the role of LUS relative to CXR for patients being evaluated for COVID-19.

Conversely, LUS did have a lower specificity than CXR. As noted, 1–2 B-lines may be non-pathologic; however, only one patient in this study was found to have 1–2 B-lines that did in fact have COVID-19. It is possible that using LUS with only one or two B-lines to direct care for patients suspected of having COVID-19 could lead to unnecessary isolation or further medical testing. Additionally, there are other etiologies for LUS B-lines, and our results will likely be most valuable when interpreted in the clinical context of the medical evaluation.

Physicians should have an estimation of pretest probability when performing and interpreting diagnostic testing, and LUS for COVID-19 is no exception to this rule. In this population with a high prevalence of disease (as judged by RT-PCR results), a positive LUS was a good predictor of disease. Further work is necessary to better delineate how to incorporate these findings into screening for asymptomatic patients, diagnostic algorithms, and clinical management strategies.

## LIMITATIONS

Since this was a retrospective study, it is unclear why physicians chose to perform both CXR and LUS. It is also unknown whether the result of either diagnostic test affected the physician’s choice to perform the other test. Additionally, the treating physician was not blinded to the patient’s history, exam, or CXR. It is possible that knowledge of these data points would change the extent to which the physician performed their LUS. Despite this, there were a similar number of images recorded for patients with and without COVID-19.

Over half of the studies performed were performed by non-fellowship trained EPs. Further work is needed to validate these findings in a population of EPs without fellowship training. Identification of B-lines is a core skill of EPs; therefore, we anticipate the findings would be similar.

Another limitation was the use of RT-PCR for the diagnosis of COVID-19, as it likely misses some cases. Some of the tests classified as false positive may have actually been true positives. RT-PCR was chosen as the reference standard since that is what is currently used at our, and most, institutions nationally, and viral culture is not feasible at this time. Inconclusive CXRs were scored as negative, which might favor the analysis toward LUS. This was done, in accordance with STARD guidelines, because inconclusive CXRs do not provide diagnostic guidance in real time.^11^

We used B-lines in this study as a reliable marker for COVID-19. It is possible a comprehensive evaluation including pleural abnormalities and subpleural consolidations would improve the test characteristics of LUS. We chose to only include B-lines for our assessment as B-lines are already familiar to EPs and would be easier to implement. We included any number of B-lines (one or more) as abnormal; however, it has been reported 1–2 B-lines may not be pathologic. We selected this approach to maximize the sensitivity of LUS at the cost of specificity.

## CONCLUSION

This investigation provides evidence that LUS is more sensitive for the associated diagnosis of COVID-19 than CXR when excluding patients with other expected causes of B-lines. This work could have important implications where viral testing is restricted or alternative diagnostic imaging is not available. Further work may find LUS for the evaluation and care of COVID-19 patients to be of clinical benefit and may also have a role to guide testing as screening and contact tracing are expanded.

## Supplementary Information













## Figures and Tables

**Table 1:** Demographic and clinical variables of patients enrolled in study to evaluate test characteristics of lung ultrasound for coronavirus disease 2019 (COVID-19).

	Overall (N=43)	COVID-19 (+) (N=27)	COVID-19 (−) (N=16)	P-value
Demographics				
Age (years), median (IQR)	52.0 (25.0)	53.0 (20.0)	50.0 (28.5)	0.880*
Race, n (%)				< 0.001†
White	12 (27.9)	3 (11.1)	9 (56.3)	
Black	15 (34.9)	8 (29.6)	7 (43.8)	
Asian	0 (0.0)	0 (0.0)	0 (0.0)	
Other/unknown	16 (37.2)	16 (59.3)	0 (0.0)	
Ethnicity, n (%)				< 0.001‡
Hispanic	12 (27.9)	12 (44.4)	0 (0.0)	
Non-Hispanic	27 (62.8)	11 (40.7)	16 (100.0)	
Unknown	4 (9.3)	4 (14.8)	0 (0.00)	
Gender, n (%)				0.076†
Male	21 (48.8)	16 (59.3)	5 (31.3)	
Female	22 (51.2)	11 (40.7)	11 (68.8)	
BMI (kg/m^2^), mean (SD)	31.6 (8.4)	31.7 (9.0)	31.3 (7.5)	0.891§
Symptom duration at time of LUS (days), mean (SD)	5.4 (4.8)	6.0 (4.9)	4.4 (4.6)	0.311§
Diabetes, n (%)	11 (25.6)	10 (37.0)	1 (6.3)	0.033‡
Asthma, n (%)	9 (20.9)	4 (14.8)	5 (31.3)	0.257‡
Obesity, n (%)	19 (44.2)	12 (44.4)	7 (43.8)	1.000‡
Coronary artery disease, n (%)	2 (4.7)	0 (0.0)	2 (12.5)	0.133‡
COPD, n (%)	3 (7.0)	1 (3.7)	2 (12.5)	0.545‡
Vital Signs				
SpO_2_ (%), median (IQR)	96.0 (3.0)	95.0 (2.0)	96.5 (3.0)	0.082*
Temperature (°F), median (IQR)	99.1 (2.1)	99.9 (2.1)	98.3 (0.9)	0.001*
Systolic blood pressure (mmHg), mean (SD)	128.7 (20.3)	126.2 (15.5)	132.8 (26.6)	0.376§
Diastolic blood pressure (mmHg), mean (SD)	76.8 (13.3)	75.0 (12.0)	79.9 (15.3)	0.255§
Initial heart rate (bpm), mean (SD)	91.2 (18.3)	96.2 (18.4)	82.8 (15.2)	0.018§
Respiratory rate (rpm), mean (SD)	21.0 (5.5)	22.0 (6.7)	19.4 (1.6)	0.070§
Diagnostic testing				
Abnormal WBC K/μL (<4 or >11), n (%)	16 (43.2)	10 (41.7)	6 (46.2)	1.000‡
Abnormal polys K/μL (<1.8 or >7.0), n (%)	13 (35.1)	9 (37.5)	4 (30.8)	0.734‡
Abnormal lymphocytes K/μL (<1.1 or >3.5), n (%)	15 (40.5)	12 (50.0)	3 (23.1)	0.166‡
Abnormal platelets K/μL (<150 or >400), n (%)	5 (13.5)	2 (8.3)	3 (23.1)	0.321‡
Abnormal sodium mmol/L (<135 or >145), n (%)	8 (21.6)	7 (29.2)	1 (7.7)	0.216‡
Abnormal ferritin ng/ml (>109), n (%)	24 (80.0)	20 (90.9)	4 (50.0)	0.029‡
Abnormal LDH U/L (>308), n (%)	16 (51.6)	14 (63.6)	2 (22.2)	0.054‡
Abnormal D-dimer ng/mL DDU (>243), n (%)	17 (54.8)	13 (61.9)	4 (40.0)	0.441‡
Abnormal Fibrinogen mg/dL (>460), n (%)	20 (66.7)	15 (71.4)	5 (55.6)	0.431‡
Abnormal ESR mm/hr (>30), n (%)	26 (83.9)	21 (91.3)	5 (62.5)	0.093‡
Abnormal CRP mg/L (>5), n (%)	29 (90.6)	21 (91.3)	8 (88.9)	1.000‡
Abnormal Brain-Natriuretic Peptide pg/ml (>72.3), n (%)	2 (6.7)	2 (9.1)	0 (0.0)	1.000‡
Clinical results				
Type of CXR, n (%)				1.000‡
Portable	42 (97.7)	26 (96.3)	16 (100.0)	
Two-view	1 (2.3)	1 (3.7)	0 (0.0)	
Admitted, n (%)				0.092‡
Yes	31 (72.1)	22 (81.5)	9 (56.3)	
No (discharged)	12 (27.9)	5 (18.5)	7 (43.75)	
If admitted, location, n (%)				0.834‡
Floor	22 (71.0)	15 (68.2)	7 (77.8)	
IMCU	3 (9.7)	2 (9.1)	1 (11.1)	
ICU	6 (19.4)	5 (22.7)	1 (11.1)	
If admitted, transferred to ICU within 48 hours, n (%)				0.286‡
Yes	5 (16.1)	5 (22.7)	0 (0.0)	
No	26 (83.9)	17 (77.3)	9 (100.0)	
Required supplemental oxygen in ED, n (%)				0.054†
Yes	16 (37.2)	13 (48.2)	3 (18.8)	
No	27 (62.8)	14 (51.9)	13 (81.3)	
LUS images recorded, mean (SD)	6.21 (3.3)	5.93 (3.7)	6.69 (2.5)	0.472§
Ultrasound probe used, n (%)				0.069‡
Phased array	6 (14.0)	6 (22.2)	0 (0.0)	
Curvilinear	37 (86.1)	21 (77.8)	16 (100.0)	
Linear	0 (0.00)	0 (0.0)	0 (0.0)	
LUS: B-lines, n (%)				< 0.001‡
0	12 (27.9)	3 (11.1)	9 (56.3)	
1–2	4 (9.3)	1 (3.7)	3 (18.8)	
≥3	27 (62.8)	23 (85.2)	4 (25.0)	
LUS: pleural thickening, n (%)	24 (55.8)	21 (77.8)	3 (18.8)	< 0.001‡
LUS: sub-pleural consolidation, n (%)	12 (27.9)	10 (37.0)	2 (12.5)	0.158‡

* Wilcoxon rank-sum test

† Chi-squared test of independence

‡ Fisher’s exact test

§ Two-independent samples t-test

*IQR*, interquartile range; *BMI*, body mass index; *kg*, kilogram; *m^2^*, meter squared; *SD*, standard deviation; *LUS*, lung ultrasound; *COPD*, chronic obstructive pulmonary disease; *SpO_2_*, oxygen saturation; *°F*, Fahrenheit; *mmHg*, millimeters of mercury; *WBC*, white blood cell count; *K/μL*, thousands per microliter; *mmol*, millimoles; *L*, liter; *ng*, nanograms; *ml*, milliliter; *LDH*, lactate dehydrogenase; *U*, units; *DDU*, D-dimer units; *mg*, milligram; *dl*, deciliter; *polys*, polymorphonuclear leukocytes.

*ESR*, erythrocyte sedimentation rate; *mm*, millimiter; *hr*, hour; *CRP*, C-reactive protein; *mg*, milligram; *L*, liter; *PG*, picogram; *ml*, milliliter; *CXR*, chest radiograph; *IMCU*, intermediate care unit; *ICU*, intensive care unit; *ED*, emergency department; LUS, lung ultrasound; *SD*, standard deviation.

**Table 2:** Association of lung ultrasound and chest radiograph findings of COVID-19.

	Value	95% CI
Sensitivity (%)		
Lung ultrasound	88.9	71.1 – 97.0
Chest radiograph	56.3	33.2 – 76.9
Specificity (%)		
Lung ultrasound	51.9	34.0 – 69.3
Chest radiograph	75.0	50.0 – 90.3
Positive predictive value (%)		
Lung ultrasound	77.4	59.9 – 88.9
Chest radiograph	77.8	54.3 – 91.5
Negative predictive value (%)		
Lung ultrasound	75.0	46.2 – 91.7
Chest radiograph	48.0	30.0 – 66.5
Positive likelihood ratio		
Lung ultrasound	2.03	0.84 – 3.23
Chest radiograph	2.07	0.10 – 4.05
Negative likelihood ratio		
Lung ultrasound	0.20	0 – 0.43
Chest radiograph	0.64	0.32 – 0.96

*CI*, confidence interval.
