# Supplementary material for: Point-of-care Lung Ultrasound Is More Sensitive than Chest Radiograph for Evaluation of COVID-19
Source: West J Emerg Med. 2020 Jun 19;21(4):771–8. doi: 10.5811/westjem.2020.5.47743 (PMC7390587; doi:10.5811/westjem.2020.5.47743)
Supplement: Supplementary file 1 [file wjem-21-771-s001.docx]

| Inaccurate CXR | COVID-19 Result | Chest radiograph findings |
| --- | --- | --- |
| False Positive | negative | Bibasilar patchy and linear opacities |
| False Positive | negative | Hazy left basilar opacity |
| False Positive | negative | Hazy opacity at the right lung base |
|  |  |  |
| False Negative | positive | Lungs are clear |
| False Negative | positive | No consolidation, pneumothorax, or pleural effusions. |
| False Negative | positive | No consolidation, pneumothorax, or pleural effusions. |
| False Negative | positive | Retrocardiac opacity likely represents subsegmental atelectasis |
| False Negative | positive | Low lung volumes |
| False Negative | positive | lungs are clear |
| False Negative | positive | lungs are clear |
| False Negative | positive | Atelectasis |
| False Negative | positive | Low lung volumes, vascular crowding, additional disease not excluded |
| False Negative | positive | lungs are clear |
| False Negative | positive | low lung volumes, atelectasis, superimposed infection not excluded |
| False Negative | Positive | Lungs are clear |

**Appendix.** Inaccurate chest radiographs
